# Supplementary material for: Hydrogen Stable Isotopes Indicate Reverse Migration of Fall Armyworm in North America
Source: Insects. 2025 Apr 29;16(5):471. doi: 10.3390/insects16050471 (PMC12112527; doi:10.3390/insects16050471)
Supplement: Supplementary file 1 [file insects-16-00471-s001.zip › insects-3527188-supplementary.pdf]

Method S1

Validation of an alternative wing-cleaning method for lepidoptera

A common method for cleaning insect wings involves rinsing them three times with a 2:1 solution of chloroform and methanol, followed by air-drying and storage until analysis [1,2]. Although the chloroform:methanol mixture is widely used for wing cleaning, we considered alternative methods to reduce potential environmental and health risks, while maintaining sample integrity and analytical accuracy.

To evaluate alternative cleaning techniques, the species *Helicoverpa zea* (Lepidoptera) was used. Eggs bought from Benzon company were reared in individual cups with artificial diet, using the same water source across all treatments to ensure consistency. We applied six treatments, combining different cleaning solvents and the removal of wing scales (referred to as "no-scales" or "NS"). The treatments included: (1) Control, with no scale removal or solvent exposure; (2) NS only; (3) Chloroform + NS; (4) Ethanol + NS; (5) 2:1 chloroform:ethanol mixture + NS; and (6) a 2:1 chloroform:methanol mixture + NS. Each treatment consisted of six replicates. This design allowed us to assess the effects of scale removal and solvent exposure on isotopic values.

After separating the wings from the thorax of each moth, they were processed according to their assigned treatment. Wings in the Control and No-scales (NS) treatments were not exposed to any solvent; scales were removed only in the NS group. For solvent treatments, all wings had their scales removed prior to cleaning. Wings assigned to the Chloroform + NS, Chloroform:Ethanol + NS, and Chloroform:Methanol + NS treatments were rinsed three times in their respective solvent solutions [1,2]. Wings in the Ethanol + NS treatment were shaken for 10 seconds and then left immersed in 70% ethanol for 24 hours. After cleaning, all wings were air-dried and stored until isotopic analysis [1].

Our results showed that the presence of scales and the absence of solvent cleaning can significantly influence the hydrogen isotope ratio (GLM: $\chi^2 = 98.8$, $p < 0.001$; Fig. S1a). All treatments involving scale removal and solvent application yielded similar results (Fig. S1a).

Samples from delta traps

Since the hydrogen isotope values obtained using 70% ethanol were statistically similar to those from the traditional 2:1 chloroform:methanol method for cleaning Lepidoptera wings, we tested a new method that incorporated Goo and Adhesive Remover Spray Gel (Goo Gone,

CC Holdings, Inc.) prior to immersing the wings in 70% ethanol. This method was applied to *Spodoptera frugiperda* (Lepidoptera). This additional step was added to remove adhesive residues from sticky delta traps commonly used to capture moths, as well as to aid in scale removal. After treatment with Goo Gone, the wings were processed following the same protocol as described for the 70% Ethanol + NS method. We then compared this treatment with both the NS and Chloroform:Methanol + NS treatments. We used six replicates per treatment.

The absence of solvent cleaning significantly affected the hydrogen isotope ratio (GLM: $\chi^2 = 45.9$, $p < 0.001$; Fig. S1b). However, no significant difference in hydrogen isotope values was observed between the treatment that included Goo Gone prior to the 70% ethanol + NS method and the traditional 2:1 chloroform:methanol method (Fig. S1b).

Table S1. Description of treatments used to clean moth wings prior to stable isotope analysis, including presence of scales, solvent type, and cleaning method. All wings were air-dried after solvent application method. NS – No scales, GG – Goo Gone.

Method	Scales	Solvent	Solvent method
Helicoverpa zea			
Control	Yes	No	-
No-scales (NS)	No	No	-
Chloroform + NS	No	Chloroform	Wings were rinsed three times
Ethanol + NS	No	Ethanol 70%	Wings were shaken for 10 sec and immersed for 24h
Chloroform:Ethanol + NS	No	2:1 Chloroform:Ethanol	Wings were rinsed three times
Chloroform:Methanol + NS	No	2:1 Chloroform:Methanol	Wings were rinsed three times
Spodoptera frugiperda			
No-scales (NS)	No	No	-
Chloroform:Methanol+NS	No	2:1 Chloroform:Methanol	Wings were rinsed three times
GG+Ethanol + NS	No	Goo Gone + 70% Ethanol	Scales were removed using Goo Gone and a paintbrush. The wings were then transferred to a vial containing 70% ethanol, shaken for 10 seconds, and left immersed for 24 hours

Figure S1 – Hydrogen isotopic ratios of the wings of *Helicoverpa zea* (a) and *Spodoptera frugiperda* (b) under different treatments used to clean wings prior to stable isotope analysis (see Table S1). a) GLM: $\chi^2 = 98.8$, $p<0.001$, b) GLM: $\chi^2 = 45.9$, $p<0.001$. Different letters represent significant difference based on Tukey's post hoc test ($p<0.05$). NS – No scales, GG – Goo Gone.

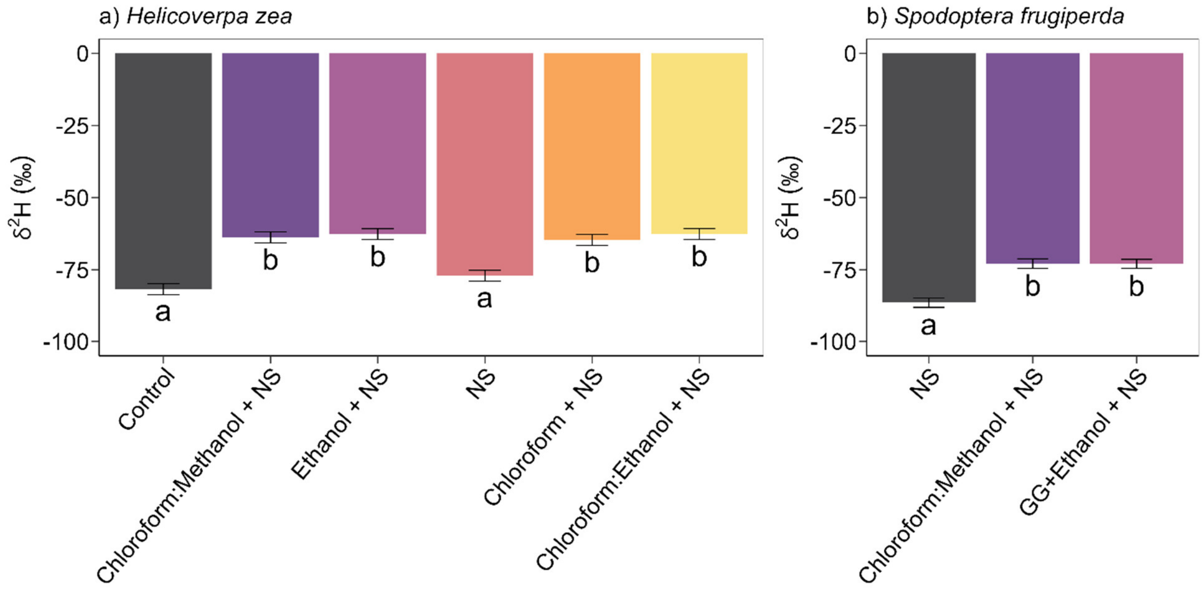

Table S2 - Hydrogen isotope ratio of 324 moths collected over 6 years during crop season in the West Florida Research and Education Center, Jay, FL.

Latitude	Longitude	Date collected	Year	δ^2H (‰)	Sample ID	Weight (mg)
30.773188	-87.14389	09/13/2018	2018	-41.0	1	191
30.773188	-87.14389	09/13/2018	2018	-52.5	2	176
30.773188	-87.14389	09/13/2018	2018	-28.5	3	166
30.773188	-87.14389	09/13/2018	2018	-36.7	4	117
30.773188	-87.14389	09/13/2018	2018	-29.0	5	148
30.773188	-87.14389	09/13/2018	2018	-51.0	6	144
30.773188	-87.14389	09/13/2018	2018	-57.8	7	118
30.773188	-87.14389	09/13/2018	2018	-38.8	8	136
30.773188	-87.14389	09/13/2018	2018	-41.1	9	98
30.773188	-87.14389	09/13/2018	2018	-33.9	10	158
30.773188	-87.14389	09/13/2018	2018	-34.7	11	110
30.773188	-87.14389	09/13/2018	2018	-46.9	12	108
30.773188	-87.14389	09/13/2018	2018	-49.2	13	145
30.773188	-87.14389	09/13/2018	2018	-57.4	14	99
30.773188	-87.14389	09/13/2018	2018	-54.4	15	177
30.773188	-87.14389	09/13/2018	2018	-60.5	16	118
30.773188	-87.14389	09/13/2018	2018	-41.7	17	173
30.773188	-87.14389	09/13/2018	2018	-52.7	18	133
30.773188	-87.14389	09/13/2018	2018	-51.9	19	101
30.773188	-87.14389	09/13/2018	2018	-65.2	20	176
30.773188	-87.14389	09/13/2018	2018	-54.9	21	136
30.773188	-87.14389	09/13/2018	2018	-46.8	22	104
30.773188	-87.14389	09/13/2018	2018	-36.0	23	145
30.773188	-87.14389	09/13/2018	2018	-32.8	24	106
30.773188	-87.14389	09/13/2018	2018	-40.1	25	123
30.773188	-87.14389	09/13/2018	2018	-42.9	26	137
30.773188	-87.14389	09/13/2018	2018	-50.0	27	114
30.773188	-87.14389	09/13/2018	2018	-42.9	28	117
30.773188	-87.14389	10/26/2018	2018	-42.8	29	116
30.773188	-87.14389	10/26/2018	2018	-49.6	30	91
30.773188	-87.14389	10/26/2018	2018	-55.2	31	175
30.773188	-87.14389	10/26/2018	2018	-51.7	32	181
30.773188	-87.14389	10/26/2018	2018	-46.0	33	165
30.773188	-87.14389	10/26/2018	2018	-51.0	34	180
30.773188	-87.14389	10/31/2018	2018	-42.5	35	88
30.773188	-87.14389	10/31/2018	2018	-33.5	36	172
30.773188	-87.14389	10/31/2018	2018	-54.9	37	125
30.773188	-87.14389	10/31/2018	2018	-61.2	38	145

30.773188	-87.14389	10/31/2018	2018	-56.5	39	110
30.773188	-87.14389	10/31/2018	2018	-54.7	40	172
30.773188	-87.14389	10/09/2018	2018	-44.7	41	137
30.773188	-87.14389	10/09/2018	2018	-30.0	42	171
30.773188	-87.14389	10/09/2018	2018	-47.2	43	169
30.773188	-87.14389	10/09/2018	2018	-30.1	44	122
30.773188	-87.14389	10/09/2018	2018	-53.8	45	115
30.773188	-87.14389	10/09/2018	2018	-23.1	46	104
30.773188	-87.14389	10/09/2018	2018	-46.6	47	139
30.773188	-87.14389	10/09/2018	2018	-32.0	48	97
30.773188	-87.14389	10/09/2018	2018	-55.2	49	152
30.773188	-87.14389	10/09/2018	2018	-56.6	50	130
30.773188	-87.14389	10/09/2018	2018	-34.0	51	101
30.773188	-87.14389	10/09/2018	2018	-60.1	52	144
30.773188	-87.14389	10/26/2018	2018	-68.6	53	136
30.773188	-87.14389	10/26/2018	2018	-62.0	54	146
30.773188	-87.14389	10/26/2018	2018	-27.6	55	91
30.773188	-87.14389	10/26/2018	2018	-50.6	56	167
30.773188	-87.14389	08/14/2020	2020	-61.7	57	138
30.773188	-87.14389	08/14/2020	2020	-68.1	58	177
30.773188	-87.14389	08/14/2020	2020	-49.8	59	125
30.773188	-87.14389	08/14/2020	2020	-50.2	60	153
30.773188	-87.14389	08/14/2020	2020	-51.4	61	136
30.773188	-87.14389	08/14/2020	2020	-50.4	62	97
30.773188	-87.14389	08/28/2020	2020	-57.6	63	147
30.773188	-87.14389	08/28/2020	2020	-39.5	64	167
30.773188	-87.14389	08/28/2020	2020	-46.3	65	129
30.773188	-87.14389	08/28/2020	2020	-62.1	66	139
30.773188	-87.14389	08/28/2020	2020	-37.8	67	106
30.773188	-87.14389	08/28/2020	2020	-42.2	68	105
30.773188	-87.14389	08/28/2020	2020	-45.9	69	138
30.773188	-87.14389	08/28/2020	2020	-92.1	70	136
30.773188	-87.14389	08/28/2020	2020	-18.1	71	141
30.773188	-87.14389	08/28/2020	2020	-62.9	72	144
30.773188	-87.14389	08/28/2020	2020	-74.0	73	129
30.773188	-87.14389	08/28/2020	2020	-46.4	74	84
30.773188	-87.14389	08/28/2020	2020	-40.0	75	94
30.773188	-87.14389	08/28/2020	2020	-51.0	76	149
30.773188	-87.14389	08/28/2020	2020	-44.1	77	105
30.773188	-87.14389	08/28/2020	2020	-41.4	78	127
30.773188	-87.14389	08/28/2020	2020	-27.5	79	139

30.773188	-87.14389	08/28/2020	2020	-37.8	80	182
30.773188	-87.14389	08/28/2020	2020	-43.6	81	116
30.773188	-87.14389	08/28/2020	2020	-39.3	82	116
30.773188	-87.14389	08/28/2020	2020	-28.2	83	101
30.773188	-87.14389	08/28/2020	2020	-46.3	84	131
30.773188	-87.14389	09/25/2020	2020	-45.9	85	117
30.773188	-87.14389	09/25/2020	2020	-47.2	86	152
30.773188	-87.14389	09/25/2020	2020	-54.5	87	132
30.773188	-87.14389	09/25/2020	2020	-40.8	88	163
30.773188	-87.14389	09/25/2020	2020	-42.9	89	165
30.773188	-87.14389	09/25/2020	2020	-36.7	90	165
30.773188	-87.14389	09/25/2020	2020	-54.8	91	105
30.773188	-87.14389	09/25/2020	2020	-53.8	92	139
30.773188	-87.14389	09/25/2020	2020	-41.7	93	130
30.773188	-87.14389	09/25/2020	2020	-55.1	94	173
30.773188	-87.14389	09/25/2020	2020	-55.4	95	164
30.773188	-87.14389	09/25/2020	2020	-44.3	96	133
30.773188	-87.14389	09/25/2020	2020	-32.7	97	146
30.773188	-87.14389	09/25/2020	2020	-61.5	98	151
30.773188	-87.14389	09/25/2020	2020	-58.8	99	150
30.773188	-87.14389	09/25/2020	2020	-42.0	100	135
30.773188	-87.14389	09/25/2020	2020	-36.5	101	140
30.773188	-87.14389	09/25/2020	2020	-48.8	102	103
30.773188	-87.14389	09/25/2020	2020	-32.7	103	96
30.773188	-87.14389	09/25/2020	2020	-24.5	104	144
30.773188	-87.14389	09/25/2020	2020	-34.2	105	183
30.773188	-87.14389	09/25/2020	2020	-32.0	106	97
30.773188	-87.14389	09/25/2020	2020	-35.3	107	144
30.773188	-87.14389	09/25/2020	2020	-40.2	108	146
30.773188	-87.14389	09/25/2020	2020	-38.8	109	110
30.773188	-87.14389	09/25/2020	2020	-51.8	110	141
30.773188	-87.14389	09/25/2020	2020	-29.4	111	167
30.773188	-87.14389	09/25/2020	2020	-32.9	112	136
30.773188	-87.14389	07/19/2023	2023	-60.0	113	170
30.773188	-87.14389	07/19/2023	2023	-52.9	114	180
30.773188	-87.14389	07/19/2023	2023	-49.4	115	165
30.773188	-87.14389	07/19/2023	2023	-51.2	116	175
30.773188	-87.14389	07/19/2023	2023	-62.7	117	123
30.773188	-87.14389	07/19/2023	2023	-49.9	118	119
30.773188	-87.14389	07/19/2023	2023	-57.2	119	143
30.773188	-87.14389	07/19/2023	2023	-47.1	120	88

30.773188	-87.14389	07/19/2023	2023	-38.8	121	146
30.773188	-87.14389	07/19/2023	2023	-52.1	122	178
30.773188	-87.14389	07/19/2023	2023	-60.0	123	178
30.773188	-87.14389	07/19/2023	2023	-35.8	124	106
30.773188	-87.14389	07/19/2023	2023	-27.8	125	128
30.773188	-87.14389	07/19/2023	2023	-45.3	126	105
30.773188	-87.14389	07/19/2023	2023	-53.8	127	135
30.773188	-87.14389	07/19/2023	2023	-52.7	128	153
30.773188	-87.14389	07/19/2023	2023	-55.0	129	122
30.773188	-87.14389	07/19/2023	2023	-52.8	130	160
30.773188	-87.14389	07/19/2023	2023	-51.4	131	100
30.773188	-87.14389	07/19/2023	2023	-13.0	132	114
30.773188	-87.14389	07/19/2023	2023	-28.4	133	147
30.773188	-87.14389	07/19/2023	2023	-38.4	134	126
30.773188	-87.14389	07/19/2023	2023	-44.3	135	158
30.773188	-87.14389	07/19/2023	2023	-39.4	136	126
30.773188	-87.14389	07/19/2023	2023	-59.8	137	155
30.773188	-87.14389	07/19/2023	2023	-32.2	138	142
30.773188	-87.14389	07/19/2023	2023	-61.6	139	155
30.773188	-87.14389	07/19/2023	2023	-74.1	140	101
30.773188	-87.14389	09/06/2023	2023	-49.2	141	118
30.773188	-87.14389	09/06/2023	2023	-26.3	142	160
30.773188	-87.14389	09/06/2023	2023	-53.6	143	103
30.773188	-87.14389	09/06/2023	2023	-24.1	144	172
30.773188	-87.14389	09/06/2023	2023	-41.9	145	149
30.773188	-87.14389	09/06/2023	2023	-57.8	146	130
30.773188	-87.14389	09/06/2023	2023	-39.1	147	144
30.773188	-87.14389	09/06/2023	2023	-36.1	148	109
30.773188	-87.14389	09/06/2023	2023	-22.2	149	93
30.773188	-87.14389	09/06/2023	2023	-24.5	150	94
30.773188	-87.14389	09/06/2023	2023	-17.1	151	145
30.773188	-87.14389	09/06/2023	2023	-22.1	152	95
30.773188	-87.14389	09/06/2023	2023	-14.0	153	136
30.773188	-87.14389	09/06/2023	2023	-29.6	154	150
30.773188	-87.14389	09/06/2023	2023	-17.4	155	139
30.773188	-87.14389	09/06/2023	2023	-31.7	156	91
30.773188	-87.14389	09/06/2023	2023	-29.1	157	135
30.773188	-87.14389	09/06/2023	2023	-4.1	158	136
30.773188	-87.14389	09/06/2023	2023	-21.8	159	123
30.773188	-87.14389	09/06/2023	2023	-29.0	160	163
30.773188	-87.14389	09/06/2023	2023	-19.5	161	164

30.773188	-87.14389	09/06/2023	2023	-25.4	162	137
30.773188	-87.14389	09/06/2023	2023	-15.6	163	115
30.773188	-87.14389	09/06/2023	2023	-49.4	164	88
30.773188	-87.14389	09/06/2023	2023	-22.9	165	106
30.773188	-87.14389	09/06/2023	2023	-29.6	166	145
30.773188	-87.14389	09/06/2023	2023	-32.8	167	112
30.773188	-87.14389	09/06/2023	2023	-29.2	168	181
30.773188	-87.14389	10/02/2019	2019	-54.6	169	132
30.773188	-87.14389	10/02/2019	2019	-52.8	170	102
30.773188	-87.14389	10/02/2019	2019	-51.3	171	127
30.773188	-87.14389	10/02/2019	2019	-45.6	172	180
30.773188	-87.14389	10/02/2019	2019	-36.5	173	172
30.773188	-87.14389	10/02/2019	2019	-36.6	174	93
30.773188	-87.14389	10/02/2019	2019	-51.0	175	130
30.773188	-87.14389	10/02/2019	2019	-34.6	176	157
30.773188	-87.14389	10/02/2019	2019	-45.7	177	158
30.773188	-87.14389	10/02/2019	2019	-49.3	178	81
30.773188	-87.14389	10/02/2019	2019	-37.7	179	117
30.773188	-87.14389	10/02/2019	2019	-34.7	180	148
30.773188	-87.14389	10/02/2019	2019	-41.4	181	162
30.773188	-87.14389	10/02/2019	2019	-42.8	182	105
30.773188	-87.14389	10/02/2019	2019	-38.5	183	89
30.773188	-87.14389	10/17/2019	2019	-8.9	184	121
30.773188	-87.14389	10/17/2019	2019	-38.9	185	138
30.773188	-87.14389	10/17/2019	2019	-53.3	186	115
30.773188	-87.14389	10/17/2019	2019	-21.5	187	135
30.773188	-87.14389	10/17/2019	2019	-36.9	188	148
30.773188	-87.14389	10/17/2019	2019	-10.5	189	169
30.773188	-87.14389	10/17/2019	2019	-23.1	190	107
30.773188	-87.14389	10/17/2019	2019	-53.0	191	123
30.773188	-87.14389	10/17/2019	2019	-36.3	192	98
30.773188	-87.14389	10/17/2019	2019	-22.1	193	164
30.773188	-87.14389	10/17/2019	2019	-33.3	194	94
30.773188	-87.14389	10/17/2019	2019	-30.1	195	89
30.773188	-87.14389	10/17/2019	2019	-51.6	196	123
30.773188	-87.14389	10/17/2019	2019	-20.0	197	96
30.773188	-87.14389	10/17/2019	2019	-64.8	198	105
30.773188	-87.14389	08/11/2021	2021	-52.6	199	100
30.773188	-87.14389	08/11/2021	2021	-57.5	200	94
30.773188	-87.14389	08/11/2021	2021	-52.4	201	135
30.773188	-87.14389	08/11/2021	2021	-59.7	202	134

30.773188	-87.14389	08/11/2021	2021	-36.0	203	88
30.773188	-87.14389	08/11/2021	2021	-41.3	204	101
30.773188	-87.14389	08/11/2021	2021	-38.2	205	138
30.773188	-87.14389	08/11/2021	2021	-39.9	206	106
30.773188	-87.14389	08/11/2021	2021	-54.6	207	116
30.773188	-87.14389	08/11/2021	2021	-59.9	208	143
30.773188	-87.14389	08/11/2021	2021	-70.8	209	128
30.773188	-87.14389	08/11/2021	2021	-47.4	210	113
30.773188	-87.14389	08/11/2021	2021	-56.2	211	159
30.773188	-87.14389	08/11/2021	2021	-53.2	212	155
30.773188	-87.14389	08/11/2021	2021	-66.2	213	111
30.773188	-87.14389	08/25/2021	2021	-43.9	214	133
30.773188	-87.14389	08/25/2021	2021	-43.7	215	91
30.773188	-87.14389	08/25/2021	2021	-39.8	216	101
30.773188	-87.14389	08/25/2021	2021	-38.1	217	92
30.773188	-87.14389	08/25/2021	2021	-53.8	218	103
30.773188	-87.14389	08/25/2021	2021	-52.1	219	122
30.773188	-87.14389	08/25/2021	2021	-58.0	220	125
30.773188	-87.14389	08/25/2021	2021	-33.2	221	165
30.773188	-87.14389	08/25/2021	2021	-43.3	222	100
30.773188	-87.14389	08/25/2021	2021	-56.3	223	143
30.773188	-87.14389	08/25/2021	2021	-45.9	224	131
30.773188	-87.14389	08/25/2021	2021	-28.9	225	110
30.773188	-87.14389	08/25/2021	2021	-59.4	226	111
30.773188	-87.14389	08/25/2021	2021	-58.2	227	147
30.773188	-87.14389	08/25/2021	2021	-46.1	228	129
30.773188	-87.14389	10/12/2022	2022	-60.4	229	153
30.773188	-87.14389	10/12/2022	2022	-50.9	230	116
30.773188	-87.14389	10/12/2022	2022	-46.5	231	116
30.773188	-87.14389	10/12/2022	2022	-31.9	232	88
30.773188	-87.14389	10/12/2022	2022	-38.8	233	109
30.773188	-87.14389	10/12/2022	2022	-32.3	234	104
30.773188	-87.14389	10/12/2022	2022	-26.8	235	170
30.773188	-87.14389	10/12/2022	2022	-44.5	236	168
30.773188	-87.14389	10/26/2022	2022	-36.3	237	94
30.773188	-87.14389	10/26/2022	2022	-42.3	238	101
30.773188	-87.14389	10/26/2022	2022	-77.3	239	133
30.773188	-87.14389	10/26/2022	2022	-47.7	240	101
30.773188	-87.14389	10/26/2022	2022	-51.0	241	125
30.773188	-87.14389	10/26/2022	2022	-57.2	242	169
30.773188	-87.14389	10/26/2022	2022	-32.8	243	84

30.773188	-87.14389	11/10/2022	2022	-29.0	244	166
30.773188	-87.14389	11/10/2022	2022	-38.1	245	139
30.773188	-87.14389	11/10/2022	2022	-34.8	246	130
30.773188	-87.14389	11/10/2022	2022	-32.8	247	162
30.773188	-87.14389	11/10/2022	2022	-23.7	248	138
30.773188	-87.14389	11/10/2022	2022	-34.0	249	107
30.773188	-87.14389	11/10/2022	2022	-36.0	250	102
30.773188	-87.14389	11/10/2022	2022	-43.1	251	115
30.773188	-87.14389	11/10/2022	2022	-38.1	252	109
30.773188	-87.14389	11/10/2022	2022	-24.6	253	162
30.773188	-87.14389	11/10/2022	2022	-42.4	254	118
30.773188	-87.14389	11/10/2022	2022	-56.9	255	154
30.773188	-87.14389	11/10/2022	2022	-42.2	256	94
30.773188	-87.14389	11/10/2022	2022	-32.8	257	93
30.773188	-87.14389	11/10/2022	2022	-35.6	258	138
30.773188	-87.14389	05/03/2018	2018	-62.4	259	174
30.773188	-87.14389	05/17/2018	2018	-38.5	260	109
30.773188	-87.14389	05/17/2018	2018	-21.3	261	91
30.773188	-87.14389	05/12/2018	2018	-60.0	262	155
30.773188	-87.14389	05/31/2018	2018	-16.3	263	172
30.773188	-87.14389	06/29/2018	2018	-51.0	264	109
30.773188	-87.14389	06/29/2018	2018	-69.5	265	147
30.773188	-87.14389	06/29/2018	2018	-63.1	266	101
30.773188	-87.14389	06/14/2018	2018	-45.5	267	126
30.773188	-87.14389	07/13/2018	2018	-44.0	268	178
30.773188	-87.14389	07/31/2018	2018	-54.6	269	109
30.773188	-87.14389	05/15/2019	2019	-78.2	270	164
30.773188	-87.14389	05/15/2019	2019	-69.2	271	154
30.773188	-87.14389	05/15/2019	2019	-54.1	272	107
30.773188	-87.14389	05/15/2019	2019	-47.7	273	105
30.773188	-87.14389	05/15/2019	2019	-57.5	274	89
30.773188	-87.14389	05/15/2019	2019	-59.9	275	93
30.773188	-87.14389	06/12/2019	2019	-34.9	276	115
30.773188	-87.14389	06/12/2019	2019	-53.3	277	89
30.773188	-87.14389	06/12/2019	2019	-47.4	278	126
30.773188	-87.14389	06/12/2019	2019	-51.2	279	124
30.773188	-87.14389	06/12/2019	2019	-65.1	280	108
30.773188	-87.14389	06/04/2020	2020	-24.9	281	117
30.773188	-87.14389	06/04/2020	2020	-59.9	282	175
30.773188	-87.14389	06/23/2020	2020	-70.8	283	96
30.773188	-87.14389	06/23/2020	2020	-66.5	284	127

30.773188	-87.14389	07/31/2020	2020	-51.6	285	145
30.773188	-87.14389	07/31/2020	2020	-66.5	286	134
30.773188	-87.14389	07/31/2020	2020	-65.5	287	104
30.773188	-87.14389	07/31/2020	2020	-64.2	288	112
30.773188	-87.14389	07/31/2020	2020	-56.5	289	135
30.773188	-87.14389	07/31/2020	2020	-31.6	290	106
30.773188	-87.14389	07/31/2020	2020	-68.5	291	100
30.773188	-87.14389	04/05/2021	2021	-56.6	292	121
30.773188	-87.14389	04/05/2021	2021	-50.6	293	105
30.773188	-87.14389	04/05/2021	2021	-36.7	294	104
30.773188	-87.14389	04/05/2021	2021	-53.0	295	93
30.773188	-87.14389	04/05/2021	2021	-22.4	296	118
30.773188	-87.14389	05/20/2021	2021	-38.1	297	175
30.773188	-87.14389	05/20/2021	2021	-59.5	298	156
30.773188	-87.14389	05/20/2021	2021	-45.9	299	91
30.773188	-87.14389	05/20/2021	2021	-64.0	300	130
30.773188	-87.14389	05/20/2021	2021	-49.0	301	154
30.773188	-87.14389	05/20/2021	2021	-65.5	302	145
30.773188	-87.14389	04/20/2022	2022	-38.7	303	157
30.773188	-87.14389	04/20/2022	2022	-60.5	304	89
30.773188	-87.14389	04/20/2022	2022	-64.7	305	116
30.773188	-87.14389	05/04/2022	2022	-43.1	306	124
30.773188	-87.14389	05/04/2022	2022	-37.0	307	114
30.773188	-87.14389	05/04/2022	2022	-61.8	308	135
30.773188	-87.14389	07/20/2022	2022	-33.7	309	94
30.773188	-87.14389	07/20/2022	2022	-23.0	310	127
30.773188	-87.14389	07/07/2022	2022	-49.8	311	95
30.773188	-87.14389	07/20/2022	2022	-33.0	312	123
30.773188	-87.14389	07/20/2022	2022	-44.6	313	120
30.773188	-87.14389	04/13/2023	2023	-41.0	314	99
30.773188	-87.14389	04/13/2023	2023	-38.9	315	135
30.773188	-87.14389	04/13/2023	2023	-67.0	316	102
30.773188	-87.14389	04/13/2023	2023	-40.6	317	113
30.773188	-87.14389	04/26/2023	2023	-72.8	318	106
30.773188	-87.14389	04/26/2023	2023	-76.4	319	168
30.773188	-87.14389	04/26/2023	2023	-68.1	320	96
30.773188	-87.14389	04/26/2023	2023	-52.0	321	146
30.773188	-87.14389	04/26/2023	2023	-67.3	322	174
30.773188	-87.14389	04/26/2023	2023	-54.9	323	97
30.773188	-87.14389	04/26/2023	2023	-61.5	324	110

References

1. Hobson, K.A.; Wassenaar, L.I.; Taylor, O.R. Stable Isotopes (δD and $\delta^{13}C$) Are Geographic Indicators of Natal Origins of Monarch Butterflies in Eastern North America. *Oecologia* **1999**, *120*, 397–404, doi:10.1007/s004420050872.
2. Dobush, G.R.; Ankney, C.D.; Krementz, D.G. The Effect of Apparatus, Extraction Time, and Solvent Type on Lipid Extractions of Snow Geese. *Can. J. Zool.* **1985**, *63*, 1917–1920, doi:10.1139/z85-285.
